# Fast Neurotransmission Related Genes Are Expressed in Non Nervous Endoderm in the Sea Anemone *Nematostella vectensis*

**DOI:** 10.1371/journal.pone.0093832

**Published:** 2014-04-04

**Authors:** Matan Oren, Itzchak Brikner, Lior Appelbaum, Oren Levy

**Affiliations:** 1 The Mina & Everard Goodman Faculty of Life Sciences, Bar-Ilan University, Ramat-Gan, Israel; 2 The Leslie and Susan Gonda Multidisciplinary Brain Research Center, Bar-Ilan University, Ramat-Gan, Israel; 3 Department of Zoology, George S. Wise Faculty of Life Sciences, Tel Aviv University, Tel Aviv, Israel; Federal University of Rio de Janeiro, Brazil

## Abstract

Cnidarian nervous systems utilize chemical transmission to transfer signals through synapses and neurons. To date, ample evidence has been accumulated for the participation of neuropeptides, primarily RFamides, in neurotransmission. Yet, it is still not clear if this is the case for the classical fast neurotransmitters such as GABA, Glutamate, Acetylcholine and Monoamines. A large repertoire of cnidarian Fast Neurotransmitter related Genes (FNGs) has been recently identified in the genome of the sea anemone, *Nematostella vectensis*. In order to test whether FNGs are localized in cnidarian neurons, we characterized the expression patterns of eight *Nematostella* genes that are closely or distantly related to human central and peripheral nervous systems genes, in adult *Nematostella* and compared them to the RFamide localization. Our results show common expression patterns for all tested genes, in a single endodermal cell layer. These expressions did not correspond with the RFamide expressing nerve cell network. Following these results we suggest that the tested *Nematostella* genes may not be directly involved in vertebrate-like fast neurotransmission.

## Introduction

The appearance of nerve cells and nerve systems is one of the most important landmarks in animal evolution, as it allowed animals to better sense and respond to their changing environment, thus improving their overall fitness. Synaptic gene elements have been identified in sponges which are the earliest known living animals that do not possess a functional nervous system [1]–[4]. The first appearance of functional nervous systems in animal evolution is attributed to the coelenterates, including the ctenophores (comb jellies) and the cnidarians [5]. Similar to higher bilaterians, cnidarian nervous systems are based on synaptic transmission [6], where neuro-signals are initiated by sensory cells in response to external cues (i.e. the cnidocytes) [7] that get transmitted through nerve cells networks resulting in muscle response [8]. The distribution of nerve cells in cnidarians is largely uniform and is frequently regarded as ‘diffuse nerve nets’ i.e. [9]. In addition, certain cnidarians have centralized nerve structures, the nerve rings, which is present in anthozoans [10], [11] and medusozoans [11]–[14] including hydrozoans [15].

Neurotransmission in cnidarians is predominated by neuropeptides [16]. A key family of neuropeptides is of the RFamids, which is characterized by a common carboxy-terminal arginine (R) and an amidated phenylalanine (F) motif [17]. Results from anatomical and functional studies show that members of the RFamides family are localized in synaptic vesicles [18], [19], and they participate in neurotransmission [5]. Due to their specific localization to a subset of cnidarian neurons, the RFamides are widely used as neuronal markers [11], [13], [20].

The cnidarian, *Nematostella vectensis*, Stephenson (1935), whose genome was sequenced [21], comprises numerous advantages as a new model organism for development and comparative studies [22]. Advantages to using this organism as a model species include ease of culture and visualization, as well as control over reproduction timing [23]. Furthermore, cnidarians are likely a sister group of bilaterians and therefore ideal for comparative neurology studies.

Results from a comprehensive bioinformatic analysis of the *Nematostella* genome indicate that there are 276 neuron-related transcripts including 110 neuropeptides and 166 nonpeptidergic Fast Neurotransmission related Genes (FNGs) of the cholinergic (n = 20), glutamatergic (n = 28), GABAnergic (n = 34) and aminergic (n = 84) systems [24].

Using whole-mount immunohistochemistry, Marlow et al. [10] localized Gamma-AminoButyric Acid (GABA) in sensory cells and neurons of *Nematostella* primary polyp. However, the results of the study showed that the expression of the Dopamine Beta Hydroxylase (DBH) orthologue do not correspond to the characterized *Nematostella* nervous system. Furthermore, the expression patterns of FNGs in adult *Nematostella* has not been shown so far, thus, it is not known whether the localization and the possible function of these genes is similar to their equivalents in the vertebrates.

Here we examined the spatial mRNA expression patterns of *Nematostella* genes that are closely or distantly related to human neuronal genes that are involved in biosynthesis, transport or degradation of classical non-peptidergic neurotransmitters, and tested whether these genes are localized in the *Nematostella* nerve/sensory cells. Our results suggest that the tested expressions are restricted to the endodermal tissue layer and are probably not localized in the adult *Nematostella* nervous system while comparing it to the *Nematostella* RFamide–positive neurons.

## Materials and Methods

### Animal maintenance

*Nematostella* individuals used in this study were bred and maintained in plastic containers with 1∶3 artificial seawater (Reef crystals) at 18°C in 12 hours light/dark regimes, in an incubator. Animals were fed (once a day, 5 days per week) with freshly hatched Artemia (brine shrimp), and their medium was renewed once a week.

### Histology

Six- to nine-month-old *Nematostella* individuals were acclimated in 7% MgCl_2_ dissolved in three volumes of FSW (Filtered Sea Water) and then fixed overnight in 4% ParaFormAldehyde (PFA), dehydrated in 70% methanol, embedded in paraffin and serially cross-sectioned (7 μm). Several paraffin sections were stained with Hematoxylin and Eosin (H&E). Other sections were used for *In Situ* Hybridization (ISH) and Immunohistochemistry.

### Gene isolation and Probe preparation

Nervous system related genes were identified using human protein sequences that were blasted (using blastp algorithm) against *N. vectensis* draft genome (http://genome.jgi-psf.org/Nemve1/Nemve1.home.html). We chose genes that their best human match was either a fast neurotransmission-related gene or gene of the same family that is not related to neurotransmission and is expressed outside the nervous system. To isolate the genes, total RNA was extracted from naïve *Nematostella* individuals using an RNeasy Mini Kit (Qiagen GmbH, Hilden, Germany; catalog no. 74104). First strand cDNA was synthesized by DNA synthesis kit (Fermentas, MD, USA; catalog no. K1621). To prepare probes for *In Situ* Hybridization (ISH) experiments, sequences were amplified by PCR (Tprofessional basic thermocycler; Biometra, Goettingen, Germany) using specially designed sets of primers as listed in Table 1. PCR products were separated on 1% agarose gel and bands of expected size were cut out for DNA isolation (QIAquick gel extraction kit; catalog no. 28704, Qiagen GmbH, Hilden, Germany). The following *Nematostella* genes were successfully isolated: NV_70014, NV_224555, NV_138860, NV_173595, NV_209664, NV_119959, NV_94865 and NV_209258 (Table 1). All PCR products were cloned into a pGEM-T-Easy vector (Promega, CA, USA catalog no. A1360) and amplified in *E-coli*. Plasmid isolation was performed with Qiagen QIAprep Spin Miniprep kit (Qiagen GmbH, Hilden, Germany catalog no. 27104) and served as a template to transcribe digoxigenin-labeled antisense mRNA probes. Sense and antisense probes (300–700 bp) were synthesized using a DIG RNA labeling kit (SP6/T7; Roche Molecular Biochemicals, Mannheim, Germany, catalog no. 11175025910).

**Table 1:** Studied *N. vectensis* Genes.

Related Neurotransmitter	Human Best Match Human Gene ID	NCBI Accession No. *Nematostella* Gene ID	Human Best Match E-value	Related Domains (E-value)	ISH Probe Primers
GABA	glutamic acid decarboxylase 2 CAB62572	XP_001632405 70014	3e-08	GadB [COG0076] (1.45e-27)	5′ GCACACCTTTGACACACATC 3′ 5′ GCTAAAGCTAAGGGCTACAAG 3′
-	glutamic acid decarboxylase 2 CAB62572	XP_001619060 224555	0.34	GadB [COG0076] (4e-20)	5′ GCGCCAGGCTTGGATCCTT 3′ 5′ GGCTTGAATTTCATGATCCATG 3′
Glutamate	vesicular glutamate transporter 3 NP_064705	XP_001623083 138860	1e-93	2A0114euk [TIGR00894] (6.60e-63)	5′ TTACCGGTGTGGAGCGTTGTCG 3′ 5′ CTCGCCCGACGCATTGATTG 3′
Glutamate	gilial high affinity glutamate transporter AAH37310	XP_001625720 173595	2e-123	SDF [pfam00375] (3.60e-88)	5′ GCCGTCAAGCATCATCTGG 3′ 5′ AGGAAATACCAAAGGCTGTGAC 3′
Acetylcholine	acetylcholinesterase AAI43470	XP_001631073 209664	4e-81	COesterase [pfam00135] (2.26e-124)	5′ TTGAGGCACTTTATAACATC 3′ 5′ GCGGTAGTCGGTTCTATG 3′
-	butyrylcholinesterase 4AQD_A	XP_001628409 119959	9e-96	COesterase [pfam00135] (2.37e-155)	5′ CTGCCATGGAAACAAGCCTG 3′ 5′ TGCTTTGGGTGTGGTTTGGATC 3′
Monoamine	monoamine oxidase A AAH44787	XP_001636466 94865	1e-55	MO [COG1231] (3.52e-30)	5′ GCGCATGTGACGACGATTC 3′ 5′ CTGGAAGTGTGGGACTGGAATC 3′
-	nicotinamide N- ethyltransferase NP_006160	XP_001631336 209258	2e-18	NNMT_PNMTTEMT [pfam01234] (1.40e-25)	5′ GGATTTGATTGGCGGCCATTC 3′ 5′ AAGCCACCAACAGCATCCTTC 3′

### *In-situ* hybridization

Organs and tissues, including body wall, tentacles, pharynx and testes were stained for whole-tissue and cell-specific expressions of eight *Nematostella* genes (Table 1).

Initially, sections were de-waxed, hydrated, post-fixated (4% PFA, 20 min.) and digested by Proteinase-K (20 μg/ml, 37°C, 20 min.). Hybridizations of probes to tissue were performed in hybridization solution containing 50% formamide, SSC X 4, 9.2 mM citric acid, 0.1% Tween 20, 50 μg/ml Heparin, 1 mg/ml denatured RNA (yeast) in 5 mM EDTA at 65°C, 6–12 hours in humid chamber. Probes were washed in formamide/SSC solutions (75%, 50%, 25% at 65°C), than in 2% SSC and twice in 0.2% SSC (65°C) and lastly in 0.2% SSC/MAB solutions (75%, 50%, 25%, pure MAB at 22°C). Sections were incubated in 1∶1000 anti-DIG-AP Antibody (3 h at 22°C) in 1% blocking solution followed by 5X15 min PBT washes. The Alkaline Phosphatase (AP) reaction was performed using BM purple and fast red AP substrates (Roche Molecular Biochemicals, Mannheim, Germany). The reaction was halted by incubation in clean tap water. Some sections were stained with DAPI (1∶500 in ddw) before mounting.

### Immunolocalization

Paraffin histological sections were pre-treated as for ISH (see above) and then washed 5X15 min in PBT, pre-incubated in 1% blocking solution and then incubated in Rabbit Anti-FMRFamide serum (Peninsula Laboratories, Europe Ltd. St. Helens, UK, IHC 8755) diluted in 1∶300 in blocking solution over night in 4°C. The following day sections were washed 5X15 min in PBT and incubated in anti-Rabbit Cy-3 or anti-Rabbit Alexa-Fluor 488 Secondary antibodies (Jackson laboratories, inc., PA, USA) until satisfactory fluorescence. Reaction was halted by incubation in clean tap water. Some sections were stained with DAPI (1∶500 in ddw) before mounting.

### Imaging

Sections were visualized and photographed using a Nikon AZ100 epifluorescent Multizoom microscope equipped with a Nikon DS-Fi1 CCD camera, Nikon DXM1200F epifluorescent microscope equipped with a Nikon eclipse 80 camera (Nikon Instech, Tokyo, Japan). Confocal imaging was performed using a Zeiss LSM710 upright confocal microscope (Zeiss, Oberkochen, Germany).

## Results

### The morphology of adult *Nematostella*

**Figure 1:**
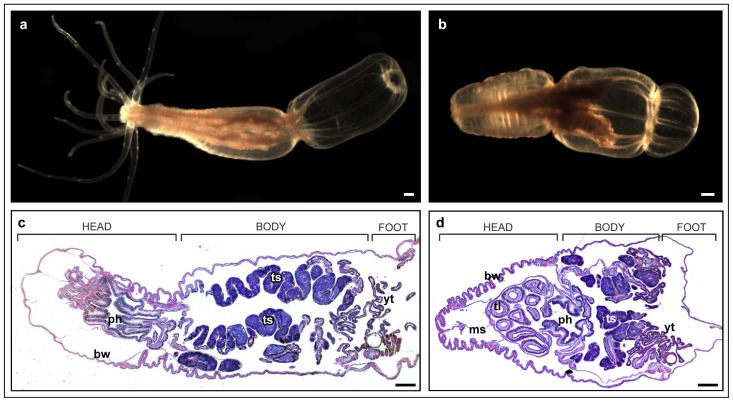
*Nematostella vectensis* morphology. (a) five months old *Nemtostella* polyp in open position with extended tentacles. (b) five months old *Nemtostella* polyp in closed position with folded tentacles inside the pharynx. (c) H&E staining of a longitudal section of *Nemtostella* polyp in open position. No tentacle tissue present. (d) H&E staining of a longitudal section of *Nemtostella* polyp in closed position. Tentacle cross-sections appear inside the pharynx cavity. Scale bars: 200 μm.

As a non-bilaterial cnidarian, *Nematostella vectensis* is diploblastic, with only two germ layers: endoderm and ectoderm. *Nematostella* polyp (Fig. 1a, 1b) is generally divided into a pharynx, mouth and tentacles-bearing head, the body cavity divided by eight mesenteries and a foot, all surrounded by the outer contractible body wall [25] (Fig. 1c, 1d). The pharynx, tentacles and the body wall are made of an out-facing ectoderm, inner-body-facing endoderm separated by the extracellular matrix of the mesoglea [25] (Fig. 1). The endodermal mesenteries bear the gonads. The maturation of the gonads in a reproductive adult is in foot to head direction, where young, pre-mature gonads are located near the foot [26] (Fig. 1c).

*Nematostella* is equipped with a sensitive nervous system that responds to changes in various parameters of its surroundings such as temperature, light and physical contact. The most indicative fast response to a change in any of these parameters is the contraction of the body to a closed position (Fig. 1b) in which the tentacles collapse into the pharynx and their circular cross-sections can be observed inside the pharyngeal cavity (Fig. 1d).

### Selection of *Nematostella* nonpeptidergic FNGs

In this study we examined eight *Nematostella* genes that were found to be closely or distantly related to genes of the GABAnergic, Glutamatergic, Cholinergic and Monoaminergic nervous sub-systems (Table 1). We tested their spatial expression patterns in comparison to RFamide-positive nervous system in *Nematostella*. The selected genes were chosen from the *Nematostella* genetic repertoire, based on best matches to human genome and previous comparative bioinformatic studies [24]. Eight corresponding sets of primers were designed (Table 1), and 300–700 bp DNA fragments were amplified from whole cDNA library and served as templates for RNA probes.

### *Nematostella* GABAnergic related gene expression

**Figure 2:**
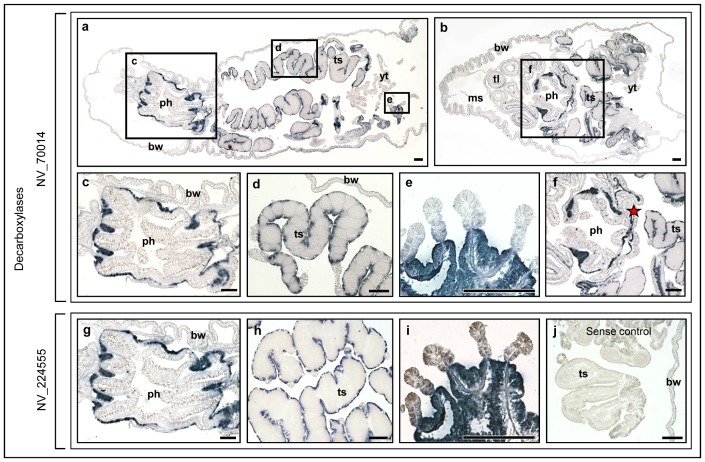
RNA expression of *N. vectensis* human glutamate decarboxilase (GAD) closely and distantly related genes. (a) *Nematostella* NV_70014 expression – whole animal longitudal section. (b) NV_70014 expression - longitudal section in a closed position. No expression is observed in tentacles. (c, g) *Nematostella* NV_70014 and NV_224555 expression showing the same localization in the endoderm around the pharynx. (d, h) NV_70014 and NV_224555 expression around the testis. (e, i) NV_70014 and NV_224555 expression in young testis. (f) NV_70014 expression - enlargement of the pharynx area showing the link between the pharyngeal expressing tissue ring and the expressing tissue surrounding gonads (marked with asterisk). (j) NV_224555 sense control with no staining. ph – pharynx, ts – testis, bw- body wall, yt – young tesis, ms – mesenteries, tl – tentacle. Scale bars: 100 μm.

GABA is the major inhibitory neurotransmitter in the vertebrate CNS. The enzyme glutamate decarboxylase (GAD) participates in GABA production and decarboxylate glutamate. Genes XP_001632403 (Nv_70014) and XP_001619060 (Nv_224555) were identified as the possible *Nematostella* GAD-like genes (table 1). While comparing these genes to the human genome (using NCBI blast) only one gene (Nv_70014) showed homology to human GAD2 while the second (Nv_224555) found to be very distantly related to this gene (Table 1). We tested the mRNA expression of the two genes using ISH. Results from the ISH analysis indicated that mRNA expression of both genes was localized in the same continuous (Fig. 2f) endodermal cell layer, which surrounds the pharynx (Fig. 2c, 2g, 2f) and testes (Fig. 2d, 2h). For testis, young testes were completely stained, indicating that there was whole-tissue expression of the two genes (2e, 2i). The genes were not expressed in tentacles (Fig 2b), body wall (Fig 2a, 2b) or any other tissue.

### *Nematostella* glutamatergic related gene expression

**Figure 3:**
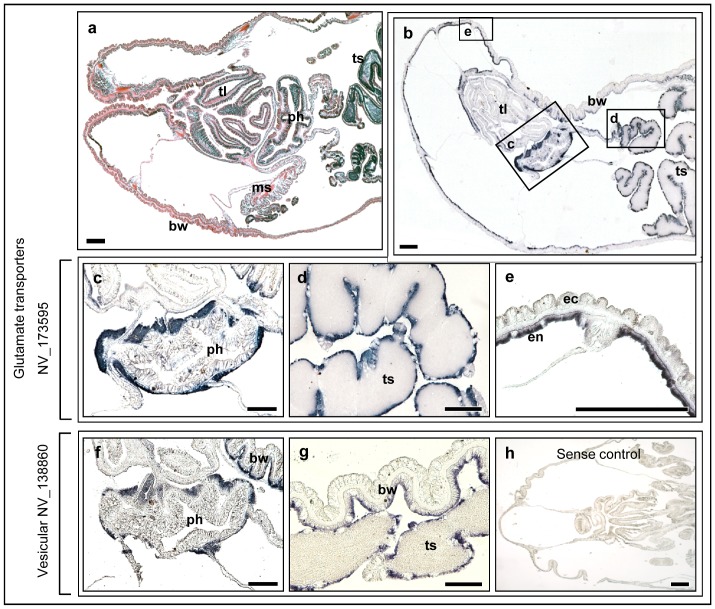
RNA expression of *N. vectensis* glutamate transporter (GLUT)-like genes. (a) H&E staining of *Nemtostella* longitudal section- head area. (b) *Nematostella* NV_173595 expression in the head and part of the body. (c, f) *Nematostella* NV_173595 and NV_138860 expression in the endoderm around the pharynx. (d) NV_173595 expression around the testis. (e) NV_173595 expression in the endoderm of the head body wall. (g) NV_138860 expression around the testis and in the endoderm of the head body wall. (h) NV_138860 sense control with no staining. ph – pharynx, ts – testis, bw- body wall, ms – mesenteries, tl – tentacle. Scale bars: 100 μm.

Glutamate is the predominant excitatory neurotransmitter in the vertebrate nervous system. In vertebrates, it is stored in chemical synapse vesicles and activates the post-synaptic nerve cells through glutamate receptors. Vertebrate glial high affinity glutamate transporter (solute carrier family 1) and vesicular glutamate transporter (vGLUT) are both excitatory amino-acid transporters (EAATs) with similar key role in regulating concentrations of glutamate in the extracellular space allowing the termination of glutamate synaptic transmission. Both vertebrate glial high affinity glutamate transporter and vGLUTs are found in glutamatergic neurons in the vertebrate CNS [27]. We have tested the expression patterns of *Nematostella* genes related to human glial high affinity glutamate transporter XP_001625720 (NV_173595) and vesicular glutamate transporter 3 XP_001623083 (NV_138860) in adult individuals. Results indicated that both genes were commonly expressed in the endodermal cell layer surrounding the pharynx (Fig. 3c, 3f) and the testis (Fig. 3d, 3g). This expression pattern is similar to *Nematostella* GAD2-like genes. However, both glutamatergic related genes were also expressed in the endoderm of the body wall surrounding the head (Fig. 3e, 3g).

### *Nematostella* cholinergic related gene expression

**Figure 4:**
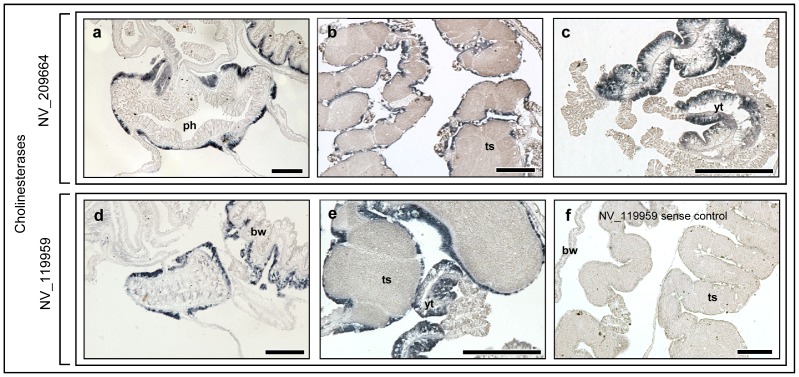
RNA expression of *N. vectensis* acetylcholinesterase (AChE) and butyrylcholinesterase (BChE). (a, d) *Nematostella* NV_209664 (AChE) and NV_119959 (BChE) expression in the endoderm around the pharynx and in the endoderm of the head body wall. (b, e) NV_209664 and NV_119959 expression in the endoderm around the testis. (c) NV_209664 expression in young testis. (f) NV_119959 sense control with no staining. ph – pharynx, ts – testis, bw- body wall, yt – young testis. Scale bars: 100 μm.

The vertebrate acetylcholine is a common neurotransmitter, which functions in both peripheral nervous systems (PNS) and CNS. It is both inhibitory (in cardiac tissue) and excitatory neurotransmitter (at neuromuscular junctions in skeletal muscle), depending on post-synaptic receptor type (reviewed in [28]). AChE is mainly found in neuromuscular junctions and cholinergic synapses of the vertebrate brain, where its activity serves to terminate synaptic transmission [29]. Here, we studied the expression patterns of *Nematostella* AChE-like XP_001631073 (NV_209664) and butyrylcholinesterase (BChE)-like XP_001628409 (NV_119959), a non-specific liver cholinesterase. Results indicated that the two genes showed similar expression patterns in the endodermal tissue around both the pharynx (Fig. 4a, 4d) and gonads (Fig. 4b, 4c, 4e), which was similar to the expression pattern of the other tested genes in these organs. Additionally, both *Nematostella* AChE and BChE were expressed in the endoderm of the head body wall (Fig. 4a, 4d), which was similar to the expression pattern of the Glutamatergic related genes in this region of the body wall.

### *Nematostella* Monoaminergic related gene expression

**Figure 5:**
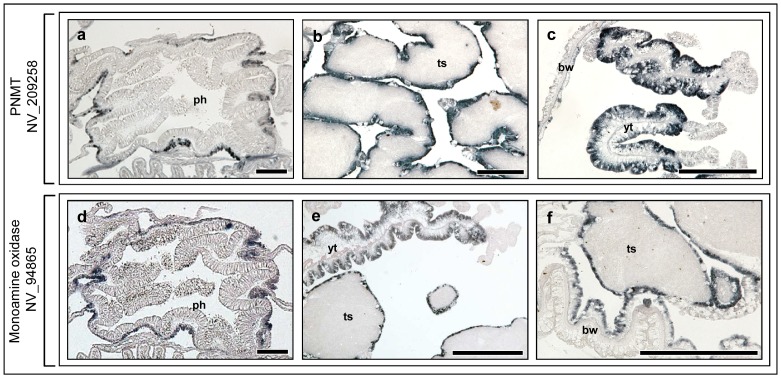
RNA expression of *N. vectensis* monoamine oxidase (MO) and nicotinamide N-methyltransferase (NNMT). (a, d) *Nematostella* NV_94865 (MO) and NV_209258 (NNMT) expression in the endoderm around the pharynx. (b, e) NV_94865 and NV_209258 expression in the endoderm around testis. Body wall endoderm is not stained. (c) NV_94865 expression in testis and in body wall endoderm. (f) NV_209258 expression in young testis. ph – pharynx, ts – testis, bw- body wall, yt – young testis. Scale bars: 100 μm.

Monoaminergic neurotransmitters are inhibitory and excitatory neurotransmitters and neuromodulators that are similar in their chemical structure but participate in many different neural pathways both inside and outside of the CNS. Monoamine oxidases (MAOs) are vital to the inactivation of monoaminergic neurotransmitters including noradrenalin (Reviewed in [30]). We have tested the expression of *Nematostella* monoamine oxidase-like XP_001636466 (NV_94865; Table 1). Results indicated that this gene is expressed in the endodermal tissue around the pharynx (Fig. 5a) and gonads (Fig. 5b, 5c) as all other tested genes. It was also expressed in the endoderm of the head body wall (Fig. 5c) as the case of *Nematostella* Glutamatergic and Cholinergic related genes. *Nematostella* gene XP_001631336 (NV_209258), with highest homology to the human non-nervous nicotinamide N-methylTransferase (NNMT) was found to have a similar expression (Fig. 5d–f) except it was not localized in the head body wall (Fig. 5f).

### FNG expressions are differently localized from *Nemtaostella* RFamide-expressing nervous system

**Figure 6:**
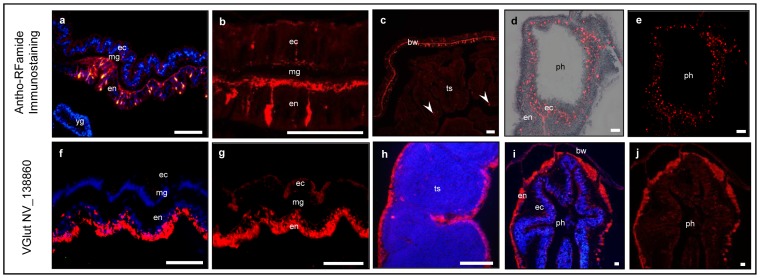
Different localization of RFamide neuropeptide and *Nematostella* vGLUT. (a–e) RFamide immunolocalization (Cy-3; red). (f–j) *Nemtostella* vGlut (NV_138860) mRNA expression (Texas red; red). (a) RFamide localization in the body wall, mostly in the basal side of the endoderm (toward the mesoglea). Cell nuclei are stained with DAPI (blue). (b) confocal microscope picture (stacking) of the body wall showing endodermal nerve-cells connected between them by a common endodermal thread. (c) Very few RFamide-positive cells (white arrow heads) were identified in the male gonads. (d) RFamide positive nerve cells scattered mostly in the ectoderm, but also in and endoderm of the pharyngeal ring. Tissue is visible in white light background. (e) Corresponding picture to d without white-light superimposing. (f) *Nemtostella* NV_138860 expression in an apical endodermal layer of the body wall. Cell nuclei are stained with DAPI (blue). (g) Corresponding picture to f without DAPI staining. (h) *Nemtostella* NV_138860 expression in the tissue surrounding the testis. Gametes are stained with DAPI (blue) (i) *Nemtostella* NV_138860 mRNA expression in the endodermal pharyngeal layer and in the epical side area of the body wall. Cell nuclei are stained with DAPI (blue). (j) corresponding picture to i without DAPI staining. ec – ectoderm, en – endoderm, ts – testis, bw – body-wall, mg – mesoglea, ph – pharynx. Scale bars: 20 μm.

*Nematostella* tested FNGs shared similar expression patterns in adult *Nematostella* histology. In order to understand whether or not FNGs are co-localized with nervous system components, we searched for a reliable marker in *Nematostella*, and found that the RFamide is a well-established marker of a subset of the nervous system in *Nematostella* and other cnidarians [11]. Therefore, we used an antibody against FMRFamide which recognizes *Nematostella* RFamide [31] to visualize *Nematostella* nerve net in our histological preparations (Fig. 6a–e) to test whether the FNGs are co-localized with nerve cells in *Nematostella*. We also performed Fluorescent *In-Situ* Hybridization (FISH), constructed a probe from the *Nematostella* vGLUT (NV_138860; Fig 6f–j) and compared the vGLUT-like expression pattern to the RFamide localization in *Nematostella* whole body sections. Results showed that the RFamide was localized in a string of highly concentrated nerve cells along the animal's body wall (Fig. 6a, 6b). This string contained large RFamide-positive nerve cell-bodies in the body wall endoderm (Fig. 6b). The extensions of these nerve cells were stretched toward the basal side of the endoderm, converging into a thick axonal thread (2–3 μM) running along the mesoglea (Fig. 6b). Fewer and smaller cell bodies were observed in the body wall ectoderm (Fig. 6a, 6b). *Nematostella* vGLUT was localized (in accordance to non-fluorescent ISH results; Fig. 3) in the apical side of the endoderm of the body wall area around the head (Fig. 6i, 6j). This expression was different than the RFamide expression, which was identified also in the body wall surrounding the tentacles, the body and the foot. In the testis, very few RFamide weakly-stained cells were localized (Fig. 6c), in contrast to the robust *Nematostella* vGLUT expression in the outer testis endoderm (Fig. 6h). Both RFamide and FNGs transcripts were localized around the pharynx cavity. However, where FNGs expression was limited to condense pharyngeal ring of endodermal cells (Fig. 6i, 6j), RFamide was localized in scattered, mostly ectodermic cells around the pharynx cavity (Fig. 6d, Fig. 6e). Our results showed that *Nematostella* vGLUT expression, as well as the expression of the other tested genes, were markedly distinct from that of RFamide suggesting that these genes are not expressed in the RFanide-positive nervous system in *Nematostella*.

## Discussion

**Figure 7:**
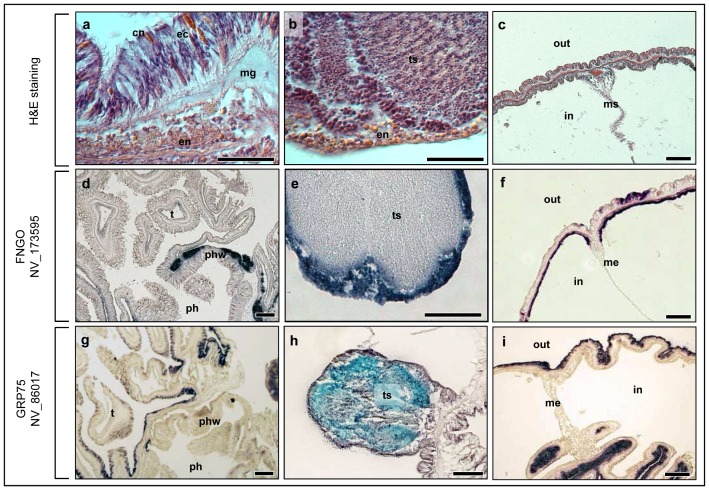
Opposite localizations of *Nematostella* GLUT-like (NV_173595) compared to *Nematostella* GRP75 (NV_86017) control. (a–c) H&E stained histology of the expressing tissues. (a) Pharynx: The tested FNGs were expressed in the pharyngeal endoderm. (b) Testis: FNGs were expressed in the endoderm surrounding the testis with same morphology and cellular composition as in a. (c) Body wall and a fragment of a mesentery in the head area: FNGs were expressed in the body wall endoderm (d–f) typical expressions of *Nematostella* FNGs as appeared for *Nematostella* GLUT (NV_173595). (d) part of the expressing endodermal tissue around the pharynx, no expression in tentacles (e) expressing endodermal tissue around the gonads (e) expressing endodermal tissue of the head body wall (g–i) expression patterns of *Nematostella* GRP75 (NV_86017). (g) No expression in pharynx and some expression in tentacles ectoderm as opposed to d. (h) GRP75 expressing gametes inside the gonads. No expression in the surrounding tissue as opposed to b. (c) strong body wall ectodermic expression as opposed to C. ec – ectoderm, en - endodem, ts – testis, cn - cnidocyte, mg- mesoglea, me-mesentery, in – interior, out – exterior. Scale bars: 40 μm.

**Figure 8:**
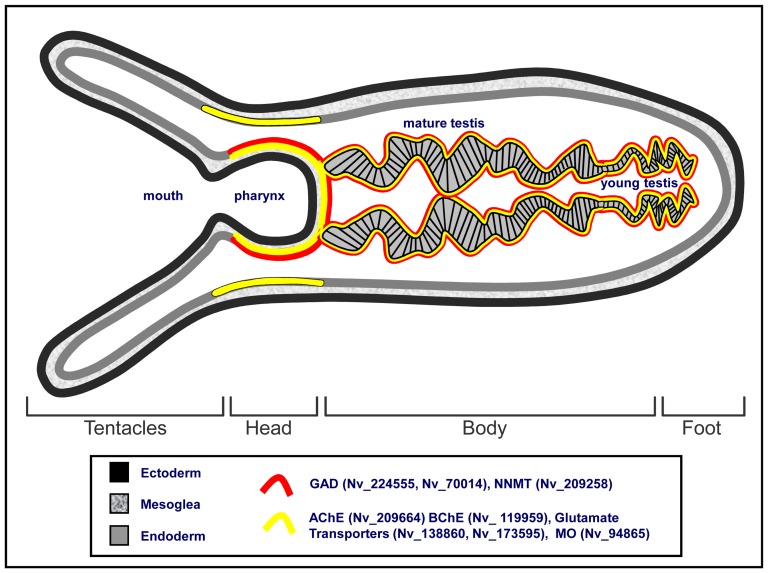
*Nematostella* FNGs expression illustration. Yellow and Red color represent localization as indicated in the legend.

In cnidarians, RFamides and other neuropeptides act as neurotransmitters and thus localize in synaptic vesicles, ganglion cells and nerve plexuses [7], [16], [32]. Non-peptidergic neurotransmitter molecules including GABA, Glutamate and Serotonin have been localized in association with the nervous system [10], [33]–[35]. Yet it is not clear whether they play an actual role in cnidarians neurotransmission [16]. Using ISH on adult *Nematostella* sections, we examined for the first time, the expression patterns of *Nematostella* genes related to human nonpeptidergic fast neurotransmission genes that are involved in biosynthesis, transport or degradation of the vertebrate's GABAnergic, glutamatergic, cholinergic and monoaminergic neurotransmitters. Our results showed common expressions for these genes as well as non-nervous more remotely associated genes in a single endodermal layer surrounding the pharynx and the testis which is 5-60 μM thick, comprised mostly of 3–5 μm round, eosinophilic cells (Fig. 7a, 7b). Shared expression was also observed in similar endodermal tissue of the head body wall, although in this region, GAD-like and NNMT-like genes were not expressed (Fig. 7c; Fig. 8). Since ISH results using adult *Nematostella* histology were rarely published, we performed ISH for *Nematostella* GRP75 (NV_86017) as a positive control to further support our technique. Results show different, sometimes opposite expression of this gene compared to the tested genes (Fig. 7d–i) re-confirming the validity of our results. The co-expression of the tested *Nematostella* genes in the same tissue (Fig. 8) may suggest common functionality. However, our expression studies suggest that FNGs role in *Nematostella* may be different than in the vertebrates since they have shared expression with genes that are likely to be non-nervous genes (i.e. BChE-like, NNMT-like) and since their expression is distinct from RFamide-positive cells. Our findings, as well as other studies (Fig. 6 a–e; [36]) suggest that RFamide is expressed in sensory and ganglionic cells of both ectoderm and endoderm. However, *Nematostella* FNGs mRNA expression is confined to a different endodermal tissue. In addition, RFamide expressing cells were abundant along the ectoderm and the endoderm of the whole body wall (Fig. 6), in the tentacles [10], around the pharynx and in low numbers in the mesenteries and the gonads (Fig. 6). In contrast, the *Nematostella* FNGs expression was limited to the head body wall endoderm (in some cases), the pharynx endoderm, around the gonads and the mesenteries (Fig. 1–7), but was not detected in the tentacles. The differences in the expression patterns of *Nematostella* FNGs and RFamide could be explained by two alternative hypothesizes. First, it is possible that the cellular location of the *Nematostella* nervous system is more elaborate than RFamide-positive nervous system. RFamide may mark only a portion of the nervous-related cells, whereby the RFamide-negative endodermal tissue may also participate in neurotransmission signaling. Supporting this is the recent identification of a distinct set of (RFamide-negative) Elav1-expressing neurons in ectoderm and endoderm [36]. These genes may also function as part of *Nematostella* nervous system and act in Trans as neuromodulators [37]. Alternatively, it is possible that the role of FNGs in *Nematostella* is markedly different than their role in higher organisms [16].

Our findings show FNGs expression around *Nematostella* gonads, a tissue which is clearly not part of the nervous system. Interestingly, GABAnergic genes including GAD and GABA receptors were found to be expressed in rodents and human testis [38], suggesting an ancient origin for this expression. An intriguing possibility was recently raised that the nervous system of the ctenophora, a sister phylum to the cnidaria, may have evolved independently from the bilaterian lineage [39]. While this may not be the case for Cnidaria, differences in the basic structure and function of the nervous system between Cnidaria and the bilaterians have been recorded. For example, neurons are derived from both ectodermal and endodermal germ layers in *Nematostella* [36]. In contrast, the neurons of bilaterians typically originate in the ectoderm layer, where the nervous system is formed [36]. Current data suggest that differences between cnidarian and bilaterians also exist in the way neurosignals are transmitted. Here we demonstrated that several *Nematostella* genes that are related to some of the key players in the vertebrate fast neurotransmission processes are not expressed in RFamide-positive neurons in *Nematostella* and therefore may not play a role in this type of neurotransmission. Further study is needed to determine whether the common spatial expression patterns of these genes reflect common functionality which is related to other types of nervous activity.
