# Correction: Fast Neurotransmission Related Genes Are Expressed in Non Nervous Endoderm in the Sea Anemone *Nematostella vectensis*

**DOI:** 10.1371/journal.pone.0103374

**Published:** 2014-07-17

**Authors:** The PLOS ONE Staff

The second author's name is spelled incorrectly. The correct name is: Itzchak Brickner. The correct citation is: Oren M, Brickner I, Appelbaum L, Levy O (2014) Fast Neurotransmission Related Genes Are Expressed in Non Nervous Endoderm in the Sea Anemone Nematostella vectensis. PLoS ONE 9(4): e93832. doi:10.1371/journal.pone.0093832

The affiliation for the third author is incorrect. Lior Appelbaum is affiliated with both #1 The Mina & Everard Goodman Faculty of Life Sciences, Bar-Ilan University, Ramat-Gan, Israel and #2 The Leslie and Susan Gonda Multidisciplinary Brain Research Center, Bar-Ilan University, Ramat-Gan, Israel.
